# The impact of frailty on clinical outcomes among older adults with diabetes: A systematic review and meta-analysis

**DOI:** 10.1097/MD.0000000000038621

**Published:** 2024-06-28

**Authors:** Min Cheng, Mei He, Liping Ning, Haoyue Gan, Qin Liu, Hangcheng Liu, Feifei Shi, Ying Luo

**Affiliations:** a School of Nursing, North Sichuan Medical University, Nanchong, China; b Nursing Department of Mianyang Central Hospital/School of Medicine Affiliated to University of Electronic Science and Technology of China, Mianyang, China.

**Keywords:** adverse outcomes, diabetes, frailty, meta-analysis, older adults

## Abstract

**Background:**

Frailty has been identified as a risk factor for adverse outcomes in older adults with diabetes. This study aimed to investigate the impact of frailty on the prognosis of older adults with diabetes through a systematic review and meta-analysis, with the goal of offering insights for clinical decision-making.

**Methods:**

PubMed, Web of Science, Embase, Cochrane were systematically searched from inception to September 10th, 2023. Reviewers independently selected studies, extracted data and evaluated the quality of studies. Stata 15.1 Software was used to perform the meta-analysis. The primary outcomes of this study were mortality, hospitalization and disability, and the secondary outcomes were diabetes complications (including nephropathy, microvascular complications, macroangiopathy, cardiovascular events, hypoglycemia) and urolithiasis.

**Results:**

A total of 14 studies were included in this study, with low risk of bias and moderate to good quality. The results showed that frailty increased the risk of mortality (HR 1.91, 95% CI 1.55–2.35, *P < *.001), hospitalization (HR 2.19, 95% CI 1.53–3.13, *P < *.001), and disability in older adults with diabetes (HR 3.84, 95% CI 2.35–6.28, *P < *.001). In addition, frailty was associated with diabetes complications (including nephropathy, microvascular complications, macroangiopathy, cardiovascular events, hypoglycemia), urolithiasis.

**Conclusions:**

Frailty is an important predictor of adverse outcomes, such as mortality, hospitalization, and disability in older adults with diabetes. Accurate assessment of the frailty in older adults with diabetes can help improve the adverse outcomes of patients.

## 1. Introduction

In recent years, the global aging situation has become increasingly serious. According to the survey, 8.5% of the global population is 65 years and older, and this proportion is expected to increase to 16.7% by 2050.^[1]^ Diabetes is one of the most common chronic diseases in the elderly. According to the statistics of the International Diabetes Federation (IDF) in 2021, the prevalence of diabetes among adults worldwide is 536.6 million, accounting for 10.5%, rising to 783.2 million by 2045, the highest prevalence age is 75 to 79 years old.^[2]^ Diabetes is related to mortality, cardiovascular and cerebrovascular diseases, which bring serious harm to families and a heavy burden to society.^[3,4]^

Frailty is an age-related geriatric syndrome, it is manifested as a decrease in physiological reserve and function of multiple organ systems, resulting in a decrease in the body ability to cope with daily or acute stressors.^[5]^ According to the survey, the global prevalence of frailty is about 3.5% to 27.3%.^[6]^ And frailty increases the risk of adverse outcomes, including mortality, hospitalization, and falls.^[7]^ There is a common underlying pathophysiological mechanism between diabetes and frailty.^[8]^ In people with diabetes, oxidative stress, inflammation, and insulin resistance can damage the skeletal muscles of patients, leading to decreased muscle strength and increased risk of frailty.^[9]^ In addition, there is evidence that frailty is associated with insulin resistance and inflammation in diabetics, which increases the incidence of adverse outcomes in patients, seriously affects the quality of life of elderly patients with diabetes, and even causes depression in the elderly.^[9,10]^ This study summarized the effects of frailty on negative health outcomes in older adults with diabetes through a systematic review and meta-analysis, aiming to provide reference information for clinical application.

## 2. Methods

This systematic review and meta-analysis were performed according to the Preferred Reporting Items for Systematic Reviews and Meta-Analyses (PRISMA) statement.^[11]^ The protocol has been registered in the International Prospective Register of Systematic Reviews (PROSPERO) and the registration number is CRD42023467159. This study involves the aggregation and analysis of existing literature results, and does not involve research on humans or animals, ethical approval is not required.

### 2.1. Search strategy

PubMed, Web of Science, Embase, Cochrane were systematically searched from inception to September 10th, 2023. The search was conducted using Medical Subject Headings (MeSH) and free terms, including frailty, frailties, frailness, frailty syndrome, diabetes mellitus, diabetes insipidus, prediabetic state, glucose intolerance, gastroparesis, etc (see Supplementary Table 1, http://links.lww.com/MD/N65, which details PubMed search strategies).

### 2.2. Eligibility criteria

The inclusion criteria were as follows: patients were diagnosed with diabetes; people were included older adults aged 60 and above; cohort studies, cross-sectional studies or longitudinal studies; frailty was defined by a recognized criterion and using the established. Exclusion criteria were: the quality evaluation score of the literature is <4; studies with incomplete data; non-English language literature.

### 2.3. Study selection and data extraction

The study from database was imported into EndNote 20 for selection. The articles were selected by 2 researchers (MC and LPN) independently according to the inclusion and exclusion criteria. In case of any differences during the selection process, a third party (HYG) was consulted to settle the issue. After reading the title and abstract of the article, study that clearly did not meet the acceptance criteria was excluded. The contents of data extraction include: study author, publication year, mean age, design type of study, prevalence of frailty, sample size, assessment of frailty, follow-up time, effect measure of study, clinical outcomes of frailty, and scores of the quality grade.

### 2.4. Quality assessment

Two researchers (MC and HCL) independently used the New Castle Ottawa Scale (NOS)^[12]^ to evaluate the quality of the included studies. The NOS scale assesses the risk of bias by assessing the quality of study on 3 dimensions, including selection, comparability, and outcome. The total score is 9 points. A score of 7 to 9 is considered high-quality research, 4 to 6 is moderate-quality research, and <4 is considered low-quality research. Cross-sectional studies were evaluated using the Agency for Healthcare Research and Quality (AHRQ).^[13]^ AHRQ included 11-item checklist. A score of 8 to 11 was considered high-quality research, 4 to 7 was moderate-quality research, and <4 was considered low-quality research.

Disagreements over quality assessment were resolved by consensus, involving a third reviewer if necessary.

### 2.5. Statistical analysis

The primary outcomes of this study were mortality, hospitalization, and disability, and other data were considered as secondary outcomes. Stata 15.1 was used for statistical analysis and graph drawing. Clinical outcomes of frailty were estimated using the hazard ratio (HR) or odds ratio (OR) with 95% CI. The statistical heterogeneity between studies used Cochran *Q* and *I^2^* to examine. If the heterogeneity was small (*I^2^* < 50%), the fixed effect model was used. If the heterogeneity was large (*I^2^* > 50%), the random effect model was used. Sensitivity analysis was performed by excluding individual studies. Subgroup analysis was used to analyze heterogeneity. Funnel plot, Beck test and Egger test were used to test publication bias.

## 3. Results

### 3.1. Search results

**Figure 1.**
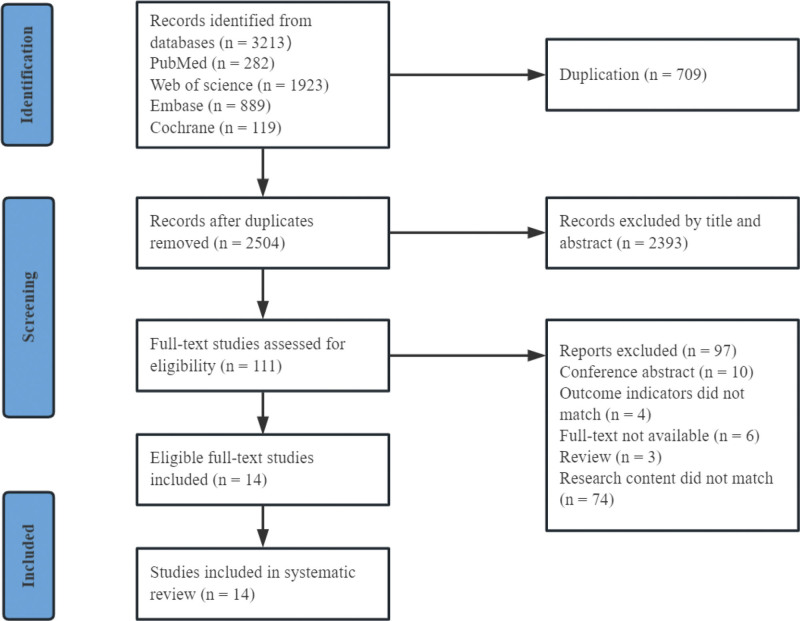
Flow diagram of study selection.

A total of 3212 studies were searched, of which 709 were duplicates. After reading the titles and abstracts of 2504 studies, 2393 studies were removed. And full-text 111 studies were read. Finally, 14 studies were included. The flow chart of study screening is shown in Figure 1.

### 3.2. Characteristics and quality assessment of included studies

A total of 14 studies were included in this study, including 13581 patients with diabetes. Studies were conducted in China,^[14–18]^ the United States,^[19–21]^ Italy,^[22]^ Spain,^[23,24]^ the United Kingdom^[25]^ and Japan.^[26,27]^ Five frailty assessment tools were used in 14 studies, including the frailty index (FI),^[14,15,19,24]^ frailty phenotype,^[20,21,26,27]^ the FRAIL,^[16–18,23]^ frailty staging system^[22]^ and clinical frailty scale (CFS).^[25]^ The characteristics and quality assessment results are shown in Table 1. Among the 14 studies, 11 were of high quality, and 3 studies were of medium quality (see Supplementary Table 2, http://links.lww.com/MD/N66 and Supplementary Table 3, http://links.lww.com/MD/N67, which details the quality scoring rules for included studies).

**Table 1:** Characteristics and quality assessment of the included studies.

Author/Yr	County	Age/years	Design	Prevalence %	N (Female%)	Frailty criteria	Follow-up	Outcomes	Quality
Ferri-Guerra 2020^[19]^	USA	72.87 ± 6.78	Cohort study	50.5%	763 (1.7%)	FI	1.53y	Hospitalization mortality	8/9
Huang 2023^[14]^	China	75.8 ± 9.8	Longitudinal study	NR	3634 (41.2%)	FI	10y	Hospitalization Mortality Cardiovascular events Hypoglycemia	8/9
Shi 2023^[15]^	China	≥60	Cohort study	17.0%	271 (NR)	FI	1y	Mortality	9/9
Chao 2020^[16]^	China	74.0 ± 12.4	Cohort study	NR	3036 (54.4%)	FRAIL	4.2y	Urolithiasis	8/9
Rodríguez 2020^[23]^	Spain	≥80	Cohort study	34.0%	199 (37.7%)	FRAIL	2y	Mortality	7/9
Leung 2021^[20]^	USA	72	Longitudinal study	25.0%	884 (50%)	FP	5y	Mortality	7/9
Kitamura 2019^[26]^	Japan	≥65	Cohort study	13.0%	176 (NR)	FP	8.1y	Mortality disability	8/9
Wang 2014^[21]^	USA	73.68 ± 5.25	Cohort study	44.0%	2415 (NR)	FP	5.6y	Mortality	7/9
Castro-Rodriguez 2016^[24]^	Spain	76	Cohort study	NR	363 (54.82%)	FI	5.5y	Mortality	8/9
Hubbard 2010^[25]^	UK	81.3	Cohort study	42.2%	310	CFS	5y	Mortality	8/9
Sable-Morita 2021^[27]^	Japan	73.8 ± 5.4	Cohort study	24.5%	477 (56.8%)	FP	2y	Neuropathy Retinopathy Nephropathy Microvascular complications	6/9
Li 2015^[17]^	China	80	Cross-sectional study	15.0%	146 (21.9%)	FRAIL	1y	Disability hospitalization macroangiopathy	7/11
Cacciatore 2013^[22]^	Italy	72.8 ± 5.8	Cross-sectional study	48.4%	188 (67.0%)	FSS	12y	Mortality	8/11
Li 2018^[18]^	China	≥65	Cross-sectional study	9.4%	719 (58.0%)	FRAIL	1y	Hospitalization	6/11

CFS = clinical frailty scale, FI = frailty index, FP = frailty phenotype, FSS = frailty staging system, NR = not reported.

### 3.3. Primary outcomes

#### 3.3.1. Mortality

**Figure 2.**
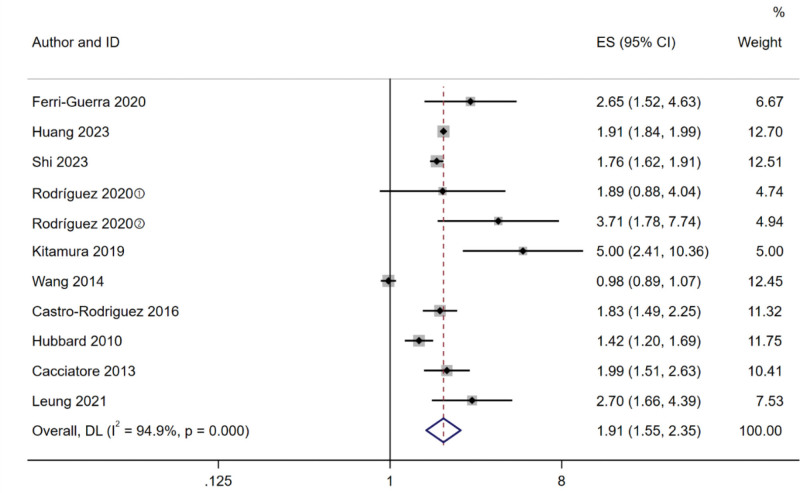
Forest plot comparing risk of mortality in older adults with diabetes between frail and non-frail patients. ① Prefrail; ② Frail.

A total of 10 studies^[14,15,19–26]^ described the relationship between frailty and mortality. After the heterogeneity test, *I^2^* = 94.9%, *P* < .001, indicating significant heterogeneity between the studies. Therefore, the random effect model was used for meta-analysis, and the results showed that the mortality rate of frailty elderly was 1.91 times higher than that of non-frailty elderly (HR 1.91, 95% CI 1.55–2.35, *P < *.001). The result is shown in Figure 2.

#### 3.3.2. Subgroup analysis

Subgroup analysis of mortality was performed based on subtypes of frailty, frailty assessment tools, region, study design, sample size, follow-up time. We divided the subtypes of frailty into pre-frailty group and frailty group, and found that the risk of mortality was lower in pre-frailty group (HR 1.89, 95% CI 0.88–4.04) than in frailty group (HR 1.91, 95% CI 1.54–2.37, *P < *.001). According to frailty assessment tools, the results showed that the CFS group had the lowest mortality (HR 1.42, 95% CI 1.20–1.69). Based on the region, the risk of mortality was lower in the Europe group (HR 1.82, 95% CI 1.45–2.28, *P < *.001) than in the America group (HR 1.85, 95% CI 0.83–4.15, *P = *.133) and Asia group (HR 1.90, 95% CI 1.66–2.17, *P < *.001). According to study design, the cohort study group had a lower risk of mortality (HR 1.89, 95% CI 1.41–2.53, *P < *.001). In the sample size, the <1000 group had a higher risk of mortality than the ≥ 1000 group. Based on the follow-up time, the risk of mortality was lower in the ≥ 2 years group (HR 1.94, 95% CI 1.46–2.58, *P < *.001). Results of subgroup analysis are shown in Table 2.

**Table 2:** Subgroup analysis of the association between frailty and mortality in older adults with diabetes.

Variables	No. of studies	*I*^2^ (%)	HR (95%CI)	*P*
Subtypes of frailty				
Frail	10	95.4	1.91 (1.54–2.37)	<.001
Prefrail	1	-	1.89 (0.88–4.04)	-
Frailty assessment tools				
FI	4	35.6	1.86 (1.74–1.98)	<.001
FRAIL	2	35.9	2.67 (1.38–5.16)	.004
FP	3	94.2	2.26 (0.83–6.12)	.110
CFS	1	-	1.42 (1.20–1.69)	-
FSS	1	-	1.99 (1.51–2.63)	-
Region				
America	3	92.6	1.85 (0.83–4.15)	.133
Asia	3	80.1	1.90 (1.66–2.17)	<.001
Europe	5	61.3	1.82 (1.45–2.28)	<.001
Study design				
Cohort study	8	94.1	1.89 (1.41–2.53)	<.001
Longitudinal study	2	48.2	2.08 (1.55–2.78)	<.001
Cross-sectional study	1	-	1.99 (1.51–2.63)	-
Sample size				
<1000	9	66.4	2.00 (1.69–2.37)	<.001
≥1000	2	99.4	1.37 (0.71–2.63)	.345
Follow-up				
<2y	2	50.6	1.96 (1.38–2.79)	<.001
≥2y	9	95.8	1.94 (1.46–2.58)	<.001

CFS = clinical frailty scale, FI = frailty index, FP = frailty phenotype, FSS = frailty staging system, HR = hazard ratio.

#### 3.3.3. Hospitalization

**Figure 3.**
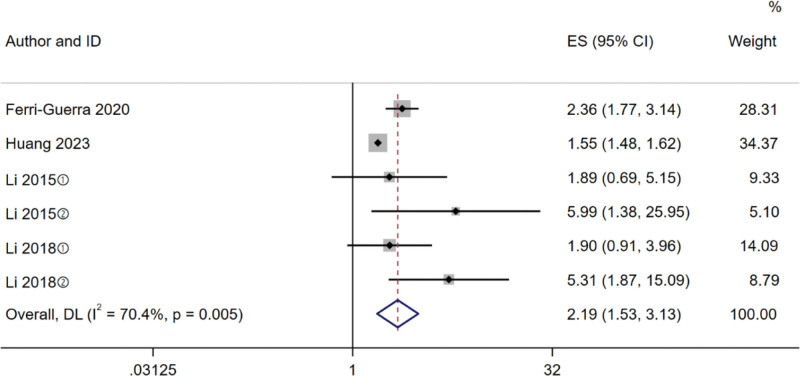
Forest plot of the association between frailty and hospitalization. ① Prefrail; ② Frail.

A total of 4 studies^[14,17–19]^ described the relationship between frailty and hospitalization, and statistical analysis showed heterogeneity among the studies (*I^2^* = 70.4%, *P* = .005), so the random effects model was selected for meta-analysis. The results showed that the incidence of hospitalization in patients with frailty was higher than that in patients without frailty (HR 2.19, 95% CI 1.53–3.13, *P < *.001). Results of subgroup analysis are shown in Figure 3. Subgroup analysis was performed according to the included subtypes of frailty, frailty assessment tools, study design, region, and sample size. According to the subtypes of frailty, the results showed that the risk of hospitalization pre-frailty group (HR 1.90, 95% CI 1.05–3.43, *P = *.034) was lower than frailty group (HR 2.38, 95% CI 1.50–3.78, *P < *.001). Based on the frailty assessment tools, FI group had the lowest risk of hospitalization (HR 1.87, 95% CI 1.24–2.81, *P = *.003). In the study design, hospitalization rates were lower in the longitudinal study group (HR 1.55, 95% CI 1.48–1.62). According to the region, the Asia group had a lower risk of hospitalization than the American group. Based on the sample size, the ≥ 1000 group had a lower risk of hospitalization. Results of subgroup analysis are shown in Table 3.

**Table 3:** Subgroup analysis of the association between frailty and hospitalization in older adults with diabetes.

Variables	No. of studies	*I*^2^ (%)	HR (95%CI)	*P*
Subtypes of frailty				
Prefrail	2	0	1.90 (1.05–3.43)	.034
Frail	4	81.8	2.38 (1.50–3.78)	<.001
Frailty assessment tools				
FI	2	87.6	1.87 (1.24–2.81)	.003
FRAIL	4	27	2.83 (1.57–5.10)	.001
Study design				
Cohort study	1	-	2.36 (1.77–3.14)	-
Longitudinal study	1	-	1.55 (1.48–1.62)	-
Cross-sectional study	4	27	2.83 (1.57–5.10)	.001
Region				
America	1	-	2.36 (1.77–3.14)	-
Asia	5	55.6	2.24 (1.37–3.67)	.001
Sample size				
<1000	5	7.3	2.47 (1.85–3.31)	<.001
≥1000	1	-	1.55 (1.48–1.62)	-

FI = frailty index, HR = hazard ratio.

#### 3.3.4. Disability

**Figure 4.**
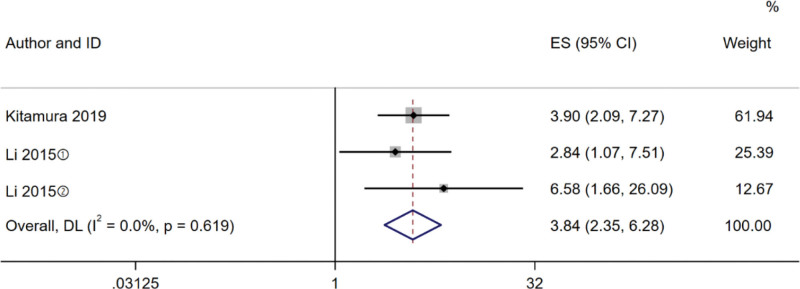
Forest plot comparing risk of disability in diabetes between frail and non-frail patients. ① Prefrail; ② Frail.

A total of 2 studies^[17,26]^ assessed the relationship between frailty and disability, and there was no heterogeneity among the studies (*I^2^* = 0%, *P* = .619), so the fixed effect model was selected for meta-analysis. The studies (Fig. 4) indicated a significantly higher incidence of disability in patients with frailty (HR 3.84, 95% CI 2.35–6.28, *P < *.001).

### 3.4. Secondary outcomes

#### 3.4.1. Diabetes complications

**Figure 5.**
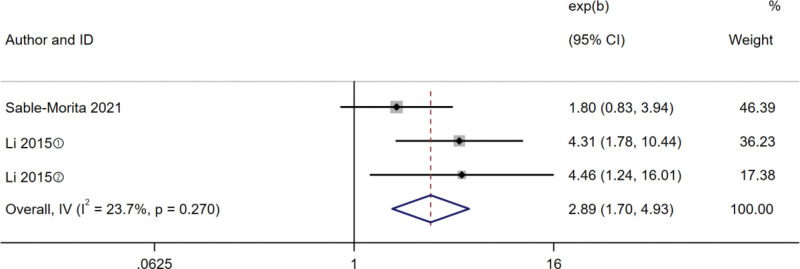
Forest plot of the relationship between frailty and nephropathy. ① Prefrail; ② Frail.

Three studies^[14,17,27]^ have described the relationship between frailty and diabetes complications, including nephropathy, microvascular complications (e.g., neuropathy, retinopathy), macroangiopathy, cardiovascular events and hypoglycemia. The results of nephropathy were combined,^[17,27]^ and the results showed that the risk of nephropathy in diabetic with frailty was 2.89 times that of diabetic without frailty (OR 2.89, 95% CI 1.70–4.93, *I*^2^ = 23.7%, *P < *.001). The result is shown in Figure 5. In addition, Sable-Morita et al^[27]^ demonstrated that frailty leaded to a higher risk of microvascular complications in diabetic patients (OR 1.426, 95% CI 1.128–1.801), with 1.225 times the risk of neuropathy (OR 1.225, 95% CI 0.621–2.416) and 1.232 times the risk of retinopathy (OR 1.232, 95% CI 0.580–2.619) in normal elderly people. However, Li et al^[17]^ showed no statistical significance between frailty and macroangiopathy in elderly patients with diabetes (OR 0.87, 95% CI 0.24–3.13, *P* = .833). Therefore, more research is needed to explore the correlation between the 2 in the future. Another study confirmed that frailty was associated with cardiovascular events (HR 1.49, 95% CI 1.42–1.57, *P* < .01) and hypoglycemia (HR 1.37, 95% CI 1.19–1.59, *P* < .01).

#### 3.4.2. Urolithiasis

Chao et al^[14]^ conducted a cohort study with a follow-up of 4.2 years, suggesting that frailty may cause urolithiasis in patients with diabetes (HR 1.46, 95% CI 1.12–1.91, *P* < .01).

### 3.5. Sensitivity analysis

**Figure 6.**
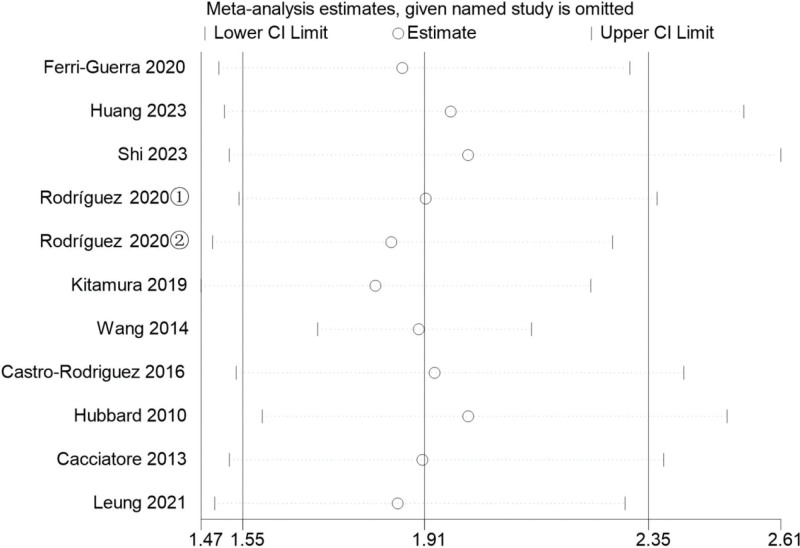
Sensitivity analysis of the effect of frailty on mortality.

A sensitivity analysis of mortality related to the main outcome variable was carried out. The random deletion of references in the study did not affect the results of the study, indicating that the calculation results of the random effects were stable. The result is shown in Figure 6.

### 3.6. Publication bias

**Figure 7.**
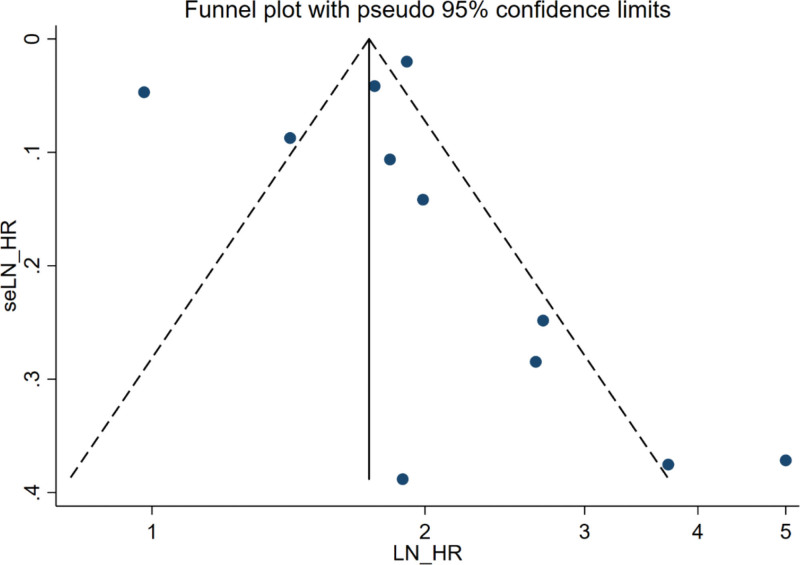
Funnel plot of the effect of frailty on mortality.

Funnel plot, Egger test and Begg test were used to test the publication bias of mortality as an outcome indicator. The results of funnel plot are shown in Figure 7. Egger test (*P* = .967) and Begg test (*P* = .350) showed no statistical significance, suggesting a small possibility of publication bias.

## 4. Discussion

A total of 14 studies were included in this study to evaluate the impact of frailty on the prognosis of older adults with diabetes, and to explore related adverse outcomes to facilitate clinical development of interventions. The results suggested that patients with diabetes had an increased incidence of adverse outcomes, including mortality, hospitalization, disability, diabetes complications (including nephropathy, microvascular complications, macroangiopathy, cardiovascular events, hypoglycemia), urolithiasis. In addition, subgroup analysis of outcome indicators mortality and hospitalization was performed in this study, and the results remained statistically significant.

Our study showed that frailty was associated with mortality in older adults with diabetes. This was consistent with the results of meta-analysis by Ida et al,^[9]^ showing that when patients with diabetes had frailty, their mortality risk increased by 1.35 times (HR 1.35, 95% CI 1.05–1.74, *P = *.02). Another meta-analysis summarizing the effect of frailty on mortality in patients with diabetes older than 18 years showed that frailty had a 1.51 times greater risk of mortality than non-frailty patients (HR 1.51, 95% CI 1.30–1.76).^[28]^ Our results showed that frailty increased the risk of mortality in older adults with diabetes by 1.91 times (HR 1.91, 95% CI 1.55–2.35, *P < *.001). This suggests that the effects of frailty are greater in older age groups. Our subgroup analysis showed no statistically significant effect of pre-frailty on the risk of mortality in older adults with diabetes. Yu et al^[29]^ showed that pre-frailty was associated with mortality in the elderly. More studies should be conducted in the future to explore the association between prefrailty and elderly patients with diabetes. The results of the subgroup analysis of this study also showed that older people with diabetes had the lowest risk of mortality in the CFS group. At present, the assessment of frailty tools is not clear, and there are differences in the assessment of frailty by different assessment tools.^[30]^ Due to the limited number of included studies, further research is needed on the impact of CFS on the assessment of older patients with diabetes. In addition, our subgroup analysis also showed that the heterogeneity between frailty and mortality was related to region. The Europe group had a lower risk of mortality. This may have something to do with eating habits. The European diet is dominated by the Mediterranean diet, study have shown that the Mediterranean diet may slow frailty, reduce the risk of age-related diseases, and reduce mortality.^[31]^

Our findings also suggested frailty may increase the risk of hospitalization in older people with diabetes. MacKenzie et al^[32]^ showed that frail elderly patients with diabetes had an increased rate of hospitalization and a longer stay. The results of subgroup analysis in this study indicated that sample size might have some impact on hospitalization. In this study, the risk of hospitalization was higher with a sample size of < 1000. When a study has a small sample size, it means that the number of participants or data points involved in the research is relatively low, which might affect the stability of results.^[33]^ In the future, studies should consider the problem of sample size in order to obtain more reliable results. In addition, the results of the subgroup analysis of hospitalization also suggested that the study design can affect the results. Due to the limitation of the number of included studies, more suitable research types can be explored in the future. The results of this study also showed that frailty was associated with the incidence of disability, and there was no heterogeneity among the studies (*I*^2^ = 0%), indicating stability in the results. The results of meta-analysis by Liu et al^[34]^ showed that the incidence of disability in frailty older adults was significantly higher than that in non-frailty older adults (RR 3.19, 95% CI 2.25–4.53) (follow-up time < 5 years). Another meta-analysis showed that frailty was an important risk factor for disability.^[35]^ Thus, performing a frailty assessment on older adults with diabetes helps to identify disability risk early and has predictive value.

In conclusion, frailty is a significant risk factor for adverse outcomes in elderly diabetic patients, who are more prone to experiencing frailty. Studies indicate that improving nutrition,^[36]^ controlling blood sugar,^[37]^ and engaging in appropriate exercise^[38]^ can improve frailty and reduce the incidence of diseases in diabetic patients. In the future, it is imperative to enhance screening for frailty in older adults with diabetes, enabling early identification and intervention to mitigate adverse outcomes.

Limitations of this study: Some outcome indicators in this study, such as disability, diabetes complications, and urolithiasis, were included in a small number of literatures, which may affect the stability of the results; Some of the study confounders were insufficiently adjusted for a possible risk of bias; The frailty assessment tools used in the included literature in this study may have affected the results; The study types were only Chinese and English literature, which may have a genre bias.

## 5. Conclusion

Through systematic review and meta-analysis, this study described that frailty is closely related to adverse outcomes such as mortality, hospitalization, disability, diabetes complications, and urolithiasis in elderly patients with diabetes. The identification, assessment and management of frailty should be part of routine older adults with diabetes care, which can help improve outcomes and improve quality of life for older people with diabetes.

## Acknowledgments

The authors wish to thank Mei He, from Mianyang Central Hospital, and Liping Ning, Haoyue Gan and Hangcheng Liu, from North Sichuan Medical College, for providing data support for this study.

## Author contributions

**Conceptualization:** Min Cheng.

**Data curation:** Min Cheng, Liping Ning, Haoyue Gan, Qin Liu, Hangcheng Liu, F Shi, Ying Luo.

**Formal analysis:** Min Cheng, Liping Ning.

**Investigation:** Min Cheng.

**Methodology:** Min Cheng, Mei He, Liping Ning.

**Project administration:** Min Cheng.

**Resources:** Min Cheng, Mei He.

**Software:** Min Cheng.

**Supervision:** Min Cheng, Mei He.

**Writing – original draft:** Min Cheng.

**Writing – review & editing:** Min Cheng.

## Supplementary Material






