# Supplementary material for: The impact of frailty on clinical outcomes among older adults with diabetes: A systematic review and meta-analysis
Source: Medicine (Baltimore). 2024 Jun 28;103(26):e38621. doi: 10.1097/MD.0000000000038621 (PMC11466167; doi:10.1097/MD.0000000000038621)
Supplement: Supplementary file 1 [file medi-103-e38621-s001.docx]

**PubMed search strategy**

#1 Frailty [MeSH]

#2 Frailties [Title/Abstract] OR Frailness [Title/Abstract] OR Frailty Syndrome [Title/Abstract] OR Debility [Title/Abstract] OR Debilities [Title/Abstract] OR frail OR pre-frail

#3 #1 OR #2

#4 Diabetes Mellitus [MeSH]

#5 Diabetes Insipidus [Title/Abstract] OR Diet, Diabetic [Title/Abstract] OR Prediabetic State [Title/Abstract] OR Scleredema Adultorum [Title/Abstract] OR Glycation End Products, Advanced [Title/Abstract] OR Glucose Intolerance [Title/Abstract] OR Gastroparesis [Title/Abstract]

#6 #4 OR #5

#7 #3 AND #6
